# Hospital and Institutional News

**Published:** 1917-08-11

**Authors:** 


### HOSPITAL AND INSTITUTIONAL NEWS.

### COLONEL CARTER.

A question was asked in the House of Commons on August 8 as to the present position of Colonel Carter, I.M.S., who was mainly responsible for the revelations of the Mesopotamia Expedition. Mr. Montagu, the Secretary of State for India, in answer, said that it was true that Colonel Carter's pay was some £300 less in this country than in India, and this was according to the regulations, but he was in communication with the Government of India in regard to the promotion of Colonel Carter, or to some other special recognition, and until he received an answer to this he could say nothing definite.

### THE INSPECTION OF MILITARY HOSPITALS.

A correspondent writes: "I have little faith in inspection. —— Military Hospital was always being inspected, and as they were sharp enough to keep things looking outwardly decent, no fault seemed to be found. How can anything to do with administration and attention to patients be seen by merely walking round with the matron and the O.C.? Inspectors only see that the place is clean and that the kits are in order. The inspector ought to go as a patient to know what a hospital is really like." The last suggestion is a counsel of perfection; but it goes to the root of the matter all the same. The only point our correspondent does not grasp is that a good inspector, even walking round in the tutelage of the matron and the O.C., can see more than that the place is clean and that the kits are in order. It depends on his capacity. If he opens cupboards he can see how far the outward appearance of cleanliness and order is superficial only. If he observes the appearance and expression of the probationers carefully he can gain hints as to how far they look or do not look worn out or overworked. In a children's hospital the mere absence or presence of crying children is a simple and infallible test. In short, an inspection is what an inspector is capable of making it. It can be a farce. But it need not be so. The military man will probably limit his inspection to an observation of the kit. The trained administrator, though not dressed in uniform, will pay more attention to vital matters. The argument, in short, is for the appointment of hospital men, rather than of military officers.

### BELGIAN CHARITY FUNDS.

Some misapprehension appears to have arisen through the recent announcement that the National Committee for Relief in Belgium had suspended appeals in this country in consequence of the United States Government taking on itself the work of relieving the Belgians in Belgium. This plainly did not mean that other Belgian charities had also stopped, and Monsieur Vandervelde, the Belgian Minister, has issued a statement to counteract a widespread impression to that effect. There are a number of useful works going on, which help our allies in several important ways; amongst these may be mentioned funds to provide comforts for the Belgian soldiers interned in Holland and their wives and families in their temporary homes, the needs of the refugees in Holland have also not been lost sight of, and there is an organisation which sends necessities to prisoners of war in Germany. In the nature of things, it is to a small extent only that those in need can rely upon their own compatriots, and, as the Minister points out, the work undertaken by the U.S.A., splendid and valuable though it is acknowledged to be, does not cover the cases we have instanced.

### A WINDFALL FOR THE LLANELLY HOSPITAL.

The Llanelly Hospital has just received a cheque for £50 under very remarkable circumstances. The principal of a South Wales steelworks was prosecuted at the Assizes at Swansea last month in connection with an alleged incorrect return in reference to output. The counsel for the defence, Mr. Llewelyn Williams, K.C., M.P., delivered a long and eloquent speech, which had such an impression upon one of the listeners in the court—a well-known Welsh coal exporter—that he went to Mr. Williams afterwards and said: "If you get the verdict I'll give you a cheque for £50 for any object you care to name." The defendant was acquitted, and Mr. Williams thereupon nominated Llanelly Hospital as the recipient of the £50, which was duly sent to the treasurer of the institution.

### THE NEW CLINIC IN GOLDEN LANE.

A pleasant instance of co-operation between a voluntary hospital and the civic authority is seen in the opening of the centre for the treatment of venereal diseases which the City Corporation has provided in Golden Lane and which is to be staffed by St. Bartholomew's Hospital. Captain Girling Ball, F.R.C.S., and Sister Fenner will be in charge. For an outlay of £1,390 a male and a female ward have been provided, together with an operating theatre, a lecture hall, waiting-rooms, an office, a dispensary, nurses' quarters, and a roof garden. The free out-patient department should be most useful, and for in- and out-patients' arrangements have been made to avoid publicity. At the opening ceremony, which was performed by Colonel W. R. Smith, M.D., Sir Thomas Barlow recalled that even in Harvey's day "Bart.s" had given special treatment to venereal cases, though mid-Victorian prudery had hindered the work of treatment, which was now being restored to its proper place. The maintenance is expected to cost £1,800, apart from a sum of £400 necessary for procuring salvarsan.

### GERMAN SOLDIERS IN WITHINGTON HOSPITAL.

The Manchester Board of Guardians have been successful in their opposition to the proposal of the military authorities to saddle them with another batch of wounded Germans for treatment at the Withington Hospital. The main building at Withington already accommodates 1,700 Germans, and when the War Office asked the board to make room for further enemy patients in the hospital annexe the Guardians declined to do so in a vigorous letter which pointed out that while so many of our own men are being treated and nursed in converted schools and similar buildings, it was unjust to devote to the Germans the special facilities of Withington, which is one of the best equipped institutions in the kingdom. The Army Council has now informed the Guardians that it will not press for the use of the annexe.

### MR. BARNES' ADMISSION.

We are glad to see that Mr. Barnes, the Pensions Minister, has repudiated on behalf of his department the practice of making inquiries at military hospitals into the earnings of men who are being examined with a view to the fixing of their disablement pensions. Mr. Montague Barlow called Mr. Barnes' attention to the practice of making these inquiries at the Wentworth Street and other Manchester hospitals, and pointedly asked why they were made, since the new warrant provided that a man's earning capacity was not to be considered in the assessment of his pension. Mr. Barnes admitted that this "irregularity" had occurred, but declared that instructions had been issued to prevent its recurrence. The Pensions Ministry constantly needs to be kept up to the mark, and Mr. Montague Barlow and other members are doing good service in subjecting Mr. Barnes to vigilant and searching examination in the House.

### THE MINISTRY OF MUNITIONS AND THE VICTIMS OF THE EXPLOSION.

We understand that the Ministry of Munitions has refused to accept the claim of the Ashton-under-Lyne District Infirmary for £550, which represents the expense incurred by the hospital in attending to the injured and dying after the serious explosion on June 13 last. The victims numbered over 100, and as the explosion occurred in a munitions factory the Ministry of Munitions has a direct interest and responsibility in the disaster. It is well to have on record the cost of the disaster, and in view of the national importance of the dangerous work on which the workers were engaged the hospital has a claim on the authorities. No doubt the Ministry is relying on the public spirit of the district or on some war charity to relieve it of its responsibility, but there must be a limit to the burdens which the charitable public is expected to shoulder, and since the War Office provides for the wounded, why should not the Ministry of Munitions do so also for the injured in the dangerous area at home?

### THE WELSH SANATORIA.

The King Edward VII. Welsh National Memorial Association has received a gift of £20,000 in War Loan Stock on condition that the number of sanatorium beds is not diminished and the scope of the work is increased. Since the gift is earmarked for the capital fund of the association it is to be used as a nucleus of a contingency fund to meet the depreciation of other securities which the war has occasioned. The institutional progress of the association during the past year has consisted in the opening of a sanatorium at Llangwyfan, near Denbigh, on a site presented by Mr. D. S. Davies; of a new hospital at Brynseiant, Carnarvon; and of Cardigan House, Newport (the gift of Sir Garrod Thomas, M.P.), where already thirty children are now under treatment. Pontywal Sanatorium, which will have 237 beds, is nearly ready, and when this is in use the association will have 1,058 beds at its disposal. On the other hand two institutions controlled by the association in Devonshire have been given up. Dr. Marcus Paterson, the medical superintendent, laments the small proportion of early cases with which the association comes into touch, and agrees with Mr. D. W. Evans, the general director, in attributing this partly to the present strain on the medical profession, and also to the strange habit of certain medical men who do not and never have referred any cases to the association, although the disease has to be notified to the medical officer of health. In short, the preventive side of the association's work is still hampered, and the general results suffer in consequence.

### A NEW WELSH HOSPITAL SCHEME.

In spite of recent expansion in the hospitals at Cardiff and in Newport, the Abertillery district is suffering from a lack of accommodation, and a provisional committee, which has a sum of some £2,500 at its disposal, is now seeing whether a temporary building cannot be erected until the permanent structure can be built. To this end the Ebbw Vale Company, which has already promised assistance, is being approached, and if labour can be obtained a temporary hospital will probably not be long delayed. Two advantages would attend its erection. First the accommodation so much desired, and second, an impetus to the fund already collected, which bricks and mortar prove to be bearing fruit. Whether, if building can be arranged, the proposed hospital can be staffed is a question which presumably has not been forgotten. At all events, the creation of local hospitals on sound lines is as good, if not better, than the creation of gigantic institutions in the big cities, where the hospitals' waiting-lists have long been a reproach to the voluntary hospital system.

### AN UNEXPECTED GIFT.

The Essex County Hospital has received a generous gift of £1,000 through Mr. W. Coates-Hutton, a trustee of a charitable purposes fund. The fund has allotted this and other gifts, and has imposed no restrictions. The financial condition of the hospital is, on the whole, satisfactory, in spite of the fact that the cost of provisions has doubled; but patients' payments, chiefly through the War Office, have shown a large increase, and there is a balance in hand on the half-year. It is to be hoped, therefore, that the above gift will be devoted to capital, as the beginning perhaps of a reserve fund to meet the depreciation of investments.

### SIR MALCOLM MORRIS AND HUDDERSFIELD INFIRMARY.

Judging by a correspondence in the local newspapers the Huddersfield Infirmary Board has been criticised by Sir Malcolm Morris and Miss Winifred Cullis for not accepting cases of venereal disease. In a spirited and courteous defence of the board's inaction Dr. P. Macgregor declares that the board is bound by by-laws, and cannot act until these have been rescinded. He adds that "the trouble is absolutely and entirely at the door of the Home Office, which apparently has tried to 'ride roughshod' over the Board's rights and susceptibilities." But Dr. Macgregor does not explain how, nor does he suggest that the board should take the purely formal steps by which the obstructive by-laws can be rescinded. Till this has been done not much faith will be excited in the board's professions. To rescind these resolutions has been a common practice of late, and we suggest that Dr. Macgregor and the board should be summoned before the bar of public opinion to show cause why they have not adopted a similar course. In making the position clear on this point Sir Malcolm Morris has done useful work, and we hope that he will continue to press his advantage.

### A VENEREAL CLINIC AT EXETER.

An out-patient department for venereal cases has been established in connection with the Royal Devon and Exeter Hospital. The house surgeon, Dr. Domville, is acting as medical officer in charge, and the board has decided wisely to appoint a fully qualified woman doctor to assist him in regard to the women patients. The new department, which is nearly in working order, is being provided at the cost of the county and city councils, which will recover from the Government three-quarters of the expense incurred. Apparently no immediate step is being taken for the treatment of cases requiring in-patient accommodation.

### HOSPITAL UNREST IN SWANSEA.

The feeling which has been aroused in Swansea through the inability of the hospital to accommodate all who seek admission, and the consequent growth of the waiting-list to nearly 200, has led to the demand that an extension must be undertaken at once. Official consent would, of course, be difficult, if not impossible, to obtain, even presumably for temporary structures, but a suggestion has been made that patients might be accommodated in a neighbouring building. On these lines, if at all, the problem may be solved; but administration under such circumstances is always a difficulty, and might lead to complaints in consequence. At the August meeting the board will disclose any expedients that seem practical, but the duration of the war cannot be pleaded indefinitely. A *modus operandi* of some kind will have to be found.

### A CHILDREN'S HOSPITAL IN DIFFICULTY.

The Bradford Children's Hospital has been placed in a disagreeable position through complaints that children sent to it quickly show symptoms of infectious disease. The fact is admitted, but the reason seems to be that the children have been infected in their own homes before admission. Once infectious disease haunts a building it is a constant source of trouble, and after a time it may be a question whether the regular arrival of infected children may not have an evil effect on the building itself. Where the cause lies can best be found with the aid of the medical officer of health, since he alone has sufficient information to determine what degree of infectious disease lurks in the different quarters of the city. The matter has become sufficiently serious to call for immediate investigation, as in the interest alike of the children and the institution, the Bradford Children's Hospital must clear itself of the prejudice which, unfortunately, has been created against it in the minds of parents in this respect.

### WAR DECREASES WORKMEN'S CONTRIBUTIONS.

The St. Austell Cottage Hospital has experienced during the past year a serious drop in its income. There were decreases in the general subscriptions, in the donations, and in the contributions from the men employed at the china clay works for which this little Cornish town is famous. The falling off in the sum given by the workmen is not due to any lack of appreciation of the work the hospital is doing, but it is accounted for by the reduction in the number of the workers consequent upon military demands. The institution owes much to the continued interest of Sir Francis Layland-Barratt, M.P., who has been elected president, and who has kindly offered to present to it after the war the *x*-ray apparatus now in use at Lady Layland-Barratt's Red Cross Hospital at Cromer. One great need of the institution is a motor ambulance to convey injured people to the hospital.

### GAS-MASKS IN WESTMINSTER HALL.

The woodworm, *Xestobium tessellatum*, has committed great depredations on the woodwork of the roof of Westminster Hall. It has destroyed quantities of wood, in some cases forming holes large enough to admit a man up to the waist. Some of the harm was done probably as long as 400 years ago, and from time to time attempts have been made to repair the damage, and to destroy the worm, but hitherto in vain. The latest attempt has been made by Mr. Frank Baines, Architect to the Office of Works; he has employed a spray, devised by Professor Maxwell Lefroy; it consists of 50 per cent. tetrachlorethane, 6 per cent. cedarwood oil, 2 per cent. soap, 2 per cent. paraffin wax, and 40 per cent. trichlorethylene. The spray is very irritating to those using it, and it was necessary for those carrying out the spraying to wear gas-masks.

### A VIVISECTION LICENCE REVOKED.

In a recent return from the Home Office particulars are given of the vivisection experiments performed during the year 1916. They amounted to 66,043, and were 4,530 less than 1915. It must be remembered that these numbers include injections, which indeed form the larger part of the total. In fact, the experiments which were not merely inoculations or injections numbered only 1,748. One of the 651 persons licensed to perform experiments injected curare without having previously obtained the special permission of the Secretary of State. He was censured, but his licence was not forfeited as he had acted from want of knowledge of the regulations. The licence of one licensee was revoked, as he had operated on an anæsthetised cat, and after the operation he had allowed the animal to regain consciousness.

### A RECORD IN PUBLIC SERVICE.

Fresh records of years spent in the public service are always coming to light, but for a man to have passed the ripe age of four score years and still to occupy such a position and fulfil his duties is worthy of record. Mr. George Taylor, who passed away—aged eighty-four—on August 4, was, we believe, the oldest coroner in England, and performed his duties until quite shortly before his death. He was a prominent citizen of Scarborough and was much associated with the affairs of the town; he was also a Justice of the Peace.

### THE CARE OF LONDON'S MENTALLY DEFICIENT.

The London County Council were appointed the administrative authority for the care of patients under the Mental Deficiency Act of 1913. It was a most unfortunate appointment, for it created another body in London to take part of the responsibilities which are still shared with the Metropolitan Asylums Board and the various Boards of Guardians, thus setting up a triple governership. Yet as the responsibility has been created, the London County Council have had to find accommodation for the patients committed to their charge, and they were faced with the problem of building, or of finding suitable premises for them. In doing this it was realised that they might be creating a second set of institutions similar to those of the Metropolitan Asylums Board, and there was consequently the danger of overlapping. For some time negotiations have taken place between the two authorities, with a view to the Metropolitan Asylums Board allowing some of the space in their institutions for the mentally defective to be used for those coming under the control of the London County Council. The latter have now under control in the Metropolis about 800 children, who have been excluded from their schools, and of these about 200 require institutional treatment. There are also a number of higher-grade cases of boys—the girls are already provided for—and a number of adult degenerate cases of both sexes, such as "in and out" workhouse cases and women of low moral character. The Metropolitan Asylums Board have a large number of available beds at their various asylums and industrial colonies for imbeciles and the feeble-minded, and they have decided to place these at the service of the London County Council, provided they can arrive at some arrangement with reference to the supply of additional information as to individual cases, but this would entail much extra work. There is no good reason why this condition should not be waived at the present time.

### LIVERPOOL AND PLAGUE.

Recently we drew attention to the report of the medical officer of the Port of Liverpool Sanitary Authority, and mentioned the precautions that were there taken to prevent the introduction of plague into the city through the port. A correspondent of the *Liverpool Journal of Commerce* brings a grave indictment against the Liverpool Port Sanitary Authority, accusing it of not adopting the most recent methods and appliances in dealing with the danger from infected rats in vessels entering the port, for traps and ratcatchers are inefficient. He claims that it is of very little use to obtain samples of rats and to examine them for evidence of plague; he urges that every rat on board a vessel should be killed if it comes from a suspected area, and that the most recent methods of destruction should be employed. Further, he points out that there is the danger that rats may escape to shore while the rats are being caught by traps and ratcatchers, for no rat-guard has yet been devised which can prevent a rat getting ashore from a vessel moored to a quay. The method of fumigating with burning sulphur is no doubt the most efficient means of ridding a ship of rats, but in Liverpool, he tells us, the method of using the sulphur vapour is to burn the sulphur in pots. The Clayton system, which is in use in the Port of London, produces large quantities of sulphurous-acid gas, and as the method is employed before the vessel comes alongside the quay, the rats have no chance of reaching the shore. Reports in favour of the Clayton system have been received from ports in many parts of the world, from Rangoon to Jamaica, and it is certain that in such an important port as Liverpool the very best methods of dealing with the rat danger should be employed. Some recent attempts have been made in America with hydrocyanic acid for the purpose of exterminating rats, but in at least two cases it has led to the death of those employing it, and therefore it hardly seems to be a convenient or suitable method.

### WATER CISTERNS.

The Metropolitan Water Board has decided that every house in the district under its control shall have a cistern, and there are certainly some arguments in favour of the decision. Indeed, to guard against periods of drought a cistern is almost a necessity. The arguments against cisterns in the house are, however, of more weight. Anyone who has had opportunities to examine the condition of many cisterns will bear witness to the unhygienic condition of a large proportion of them. As a rule the cistern is hardly ever cleaned, it is frequently left without any cover, or it possesses a cover which is far from dust-tight, so that not only dust but many other objectionable substances can find their way into it. It is not a rare occurrence to find a dead mouse in a cistern, and it is not difficult to see how many channels of risk are provided by it. The overflow pipe may also be the means of introducing septic material into a cistern; it is true that it is supposed always to discharge into the open air, but very frequently it does not. When the water supply of a closet is drawn directly from a cistern, and this is by no means so rare as many people think, the chance of the ascent of material from the closet is certainly not negligible. In many cisterns the water is stagnant and rarely is the whole cistern emptied. It is true that with due care it is possible to prevent a cistern from being a source of danger, but it is extremely rare for the requisite care to be forthcoming, and untold harm may come from a neglected cistern.

### THE LONDON MEDICAL SCHOOL SERVICE.

Not very long ago the Education Committee of the London County Council decided that, from motives of economy, it was desirable to limit the medical examination of children in the elementary schools of London, and thus it was possible to diminish the number of school medical officers, and also to provide some of the medical men needed by the War Office. The experiment was, however, hardly a success, for, although the examination of any children showing signs of ill-health was still continued, many cases of illness were missed and the general health of the children in the schools was certainly lowered and infection increased. As the result of the report of Dr. Hamer, the Medical Officer of the London County Council, the Education Committee has decided to restore to the old footing the staff of doctors available for the School Medical Service, and this change has been admitted to be urgent. It is, therefore, proposed to revert to the former arrangement as soon as possible, and every infant-entrant is to be examined in detail. When this can be carried out is by no means certain, for the supply of medical men is small, and though no doubt a certain number of medical women are available, it is doubtful whether it will be possible to obtain the full number required. The school sessions begin in September, and it will be interesting to see if the Committee will resume the examination of entrants then. The example of the London Education Committee will doubtless be followed by other educational bodies, who have also reduced the examination of school children, and therefore the effect should be good.

### SCHOOL CLINICS FOR DURHAM.

The fact that school medical work will not be thoroughly effective until school clinics are established throughout the country is strongly insisted upon in a report by Dr. T. E. Hill, the county medical officer for Durham. Having been instructed to prepare a scheme of school clinics, Dr. Hill inquired of the voluntary hospitals in the area whether they would be prepared to provide treatment for children urgently in need of attention, but as many of the institutions find it impossible under present conditions to do so, the matter will have to rest in abeyance till after the war. The County Council has approved a scheme submitted by Dr. Hill for the appointment of trained midwives in six areas where there is no satisfactory midwife practising.

### A CONVALESCENT HOME FOR MUNITION WORKERS.

The Countess of Derby has placed one of the wings of Knowsley Hall, fully furnished and equipped, at the disposal of a local committee for use as a convalescent hospital for women munition workers. The hospital is to be staffed mainly by V.A.D. workers. Knowsley was the birthplace, by the way, of many classic nonsense rhymes. Edward Lear, the inventor of the art, spent four years there during the time of the thirteenth Lord Derby, and he composed his first book of nonsense verses for the amusement of his host's children, the "inspiration" being given to him by a fellow-guest at Knowsley who chanced to give utterance to the suggestive line, "There was an old man of Tobago."

### BABY WELFARE IN ONTARIO.

An important piece of work in connection with the safeguarding of child-life in Canada is being undertaken in Ontario, where the first infant mortality survey of the Dominion is to be made. A thorough investigation will be carried out under the authority of the Provincial Board of Health at Hamilton. A record will be prepared of all the children born in 1915—the latest year for which there are statistics—and this record will enable the workers to make a study of the care, feeding, and housing conditions under which the children exist, and also of the infectious diseases to which they have been exposed. There will be about 2,800 births to investigate, and the Department intends to employ twenty-five to thirty university graduates or other women who have taken up social service work. In the province of Ontario in 1915 there were 67,032 births, and the mortality amongst these was 6,838, apart from 2,807 stillbirths.

### CHILD WELFARE IN LIVERPOOL.

The Lady Mayoress of Liverpool is throwing great energy into her efforts in connection with maternity work and child welfare, and the fund which she has inaugurated in order to supply sinews of war for the city's life-saving campaign now amounts to well over £5,000. The Lady Mayoress, who is making herself personally acquainted with the operations of agencies already existing, recently visited all the seven district homes of the Liverpool Queen Victoria District Nursing Association, and expressed great pleasure at the self-sacrifice shown by the staffs in remaining at home to look after the wives and children of so many of our sailors and soldiers.

### RAID ARRANGEMENTS.

Hunnish methods of warfare have compelled the authorities to adapt themselves to circumstances, and first-aid appliances are being kept in readiness at schools in the danger zone. Local authorities are also fitting up dressing stations so that cases may be dealt with promptly, though, if possible, they will be sent direct to hospitals. The Canning Town branch of the National Union of General Workers suggested that the West Ham Corporation should supply school children with respirators and teach them the way to use them, but the Education Committee has replied that the head teachers have full discretionary powers in the event of air-raids. London borough councils are establishing rescue and emergency organisations under the supervision of their surveyors, and some of them are compiling lists of places where people can with some degree of safety take refuge when the Huns are overhead.

### A STAFFING PROBLEM.

The problem of staffing institutions has long been acute. At West Ham the Corporation was anxious to open additional wards at its sanatorium at Dagenham, but now has to report that it is not possible to arrange any extension at present, the difficulty of obtaining the necessary staff proving insuperable. On the subject of staff troubles it may be mentioned that the London County Council cannot get officials for one of its reformatory schools, and suggests that selected boys should be appointed to act as officials. A London Poor-Law board recently had to appoint a woman as "female orderly" because it could not get a man.

### LUNATICS IN OFFICE.

Even lunatics are doing their bit during the great war. A Commissioner of the Board of Control who has paid a visit to the Birmingham Asylum records the interesting fact that for the past twelve months Dr. Roscrow has made use of the services of six trusted patients to act as assistant attendants in some of the wards. They have the use of keys and have certain privileges granted them in the matter of greater freedom, and they have in no instance abused their trust, but have proved most useful in the present depleted condition of the male staff.

### EMERGENCY MEDICINE.

The General Medical Council has met the difficulties occasioned by the sugar shortage and the embargo on glycerine in a way that is drastic to say the least of it. According to a notice published in the *London Gazette* of July 27 the Council, in pursuance of the absolute powers vested in them, have decreed that until further notice a long list of compounds which are composed partly of sugar or glycerine shall cease to be included among the official preparations of the British Pharmacopœia. The necessity for meddling with Pharmacopœial standards was presumably urgent, but it is at least unfortunate that while tens of thousands of shops are well stocked with sweetmeats composed almost entirely of sugar, the comparatively small amount of sugar required to maintain the standards of the Pharmacopœia should not be available. The Council's action is likely to cause no little confusion. A pharmacist who holds a stock of preparations made in accordance with the official formulæ, or is able to replenish his stocks, will no doubt continue to dispense medicines as before; on the other hand, a neighbouring pharmacist less happily situated may be obliged to resort to the modified preparations. What will be the result? A patient takes his prescription to the sugar chemist and has it dispensed properly; when the bottle is empty he takes the prescription to the sugarless chemist, who dispenses something that tastes quite different; result, the patient thinks he is poisoned. Or again, let us suppose the doctor orders compound liquorice powder in teaspoonful doses; one chemist supplies the Pharmacopœia preparation; the other leaves out the sugar. The result is that the patient with the sugarless compound takes twice as much senna and twice as much liquorice-root as the doctor intended! Clearly it is essential that pharmacists should come to an early agreement as to uniformity of practice. Would it not have been better had the General Medical Council fixed a date after which no sugar preparation made according to the Pharmacopœia should be dispensed, and laid down definite formulæ for use after that date? So far as hospital practice is concerned, the amount of inconvenience is not likely to be so considerable as it will be in general practice.

### THIS WEEK'S DRUG MARKET.

Owing to the intervention of Bank Holiday business has naturally been quiet; in fact, at the time of writing it is not fully resumed. Price changes of importance have been few in number, but there is no sign that the upward tendency of values is likely to receive a check in the near future. Notwithstanding that stocks of quinine in London have been greatly reduced, the advance in price is slight and buying is restricted to a small volume. A further rise in the quotations for strychnine would not come as a surprise, as the difficulty in obtaining the raw material, nux vomica, is increasing. The value of potassium bromide is maintained at the recent advance. Consequent upon the further advance in the cost of opium, makers of codeine have raised their quotations; the price of morphine hydrochloride, however, is unchanged. English refiners of camphor are offering bells and flowers more freely, but they are unable to offer tablets. Olive oil is likely to become much scarcer owing to the fact that Spain as well as Italy have prohibited the export of the oil; prices will probably advance. Atropine is scarcer and the price is higher. Chloral hydrate is slightly dearer. Acetanilide is more freely offered, and the value has a slightly downward tendency; benzoic acid is also a little cheaper. Menthol is dearer. The harvesting of the English medicinal herb crops was interfered with by the recent heavy rains, and it is feared that no little damage was done.

### TO OUR READERS.

**Contributions are specially invited from any of our readers to these columns. They should deal with topical subjects and news. They must be authenticated for the information of the Editor only. The minimum payment if published is 5s.** There is no hard-and-fast rule as to space, but notes of about twenty lines in length are preferred.

